# Hazard and determinants of dropout and rehospitalization in patients with obesity after residential rehabilitation

**DOI:** 10.1007/s40618-025-02708-z

**Published:** 2025-09-27

**Authors:** Daniele Sola, Samuele Minari, Raffaella Sabatino, Davide Soranna, Elisa Prina, Stefania Mai, Silvia Martinelli, Roberta Vietti, Raffaella Radin, Alessandra Rimella, Antonella Zambon, Massimo Scacchi

**Affiliations:** 1 Laboratory of Metabolic Research, IRCCS Istituto Auxologico Italiano, Oggebbio, Italy; 2 Department of Translational Medicine, Università del Piemonte Orientale, Novara, Italy; 3 Department of Medicine and Surgery, Università di Milano-Bicocca, Milano, Italy; 4 Molecular Ecology Group (MEG), National Research Council of Italy (CNR), Water Research Institute (IRSA), Verbania, Italy; 5 IRCCS Istituto Auxologico Italiano, Biostatistics Unit, Milano, Italy; 6 Laboratory of Clinical Neurobiology, IRCCS Istituto Auxologico Italiano, Oggebbio, Italy; 7 Laboratory of Nutrition and Obesity Research, Department of Endocrine and Metabolic Diseases, IRCCS Istituto Auxologico Italiano, Milano, Italy; 8 Private nutritional office, Verbania, Italy; 9 SSD Diabetology and Endocrinology, ASST Valle Olona, Gallarate, Italy; 10 Department of Statistics and Quantitative Methods, Università di Milano-Bicocca, Milano, Italy; 11 Department of Clinical Sciences and Community Health, Dipartimento di eccellenza 2023-2027, Università di Milano, Milano, Italy; 12 Ferrini Franzosini Institute, Verbania, Italy

**Keywords:** Obesity, Metabolic rehabilitation, Weight-loss treatment, Hazard, Dropout, Re-hospitalization

## Abstract

**Purpose:**

To identify clinical and sociodemographic factors that predict follow-up discontinuation and rehospitalisation after multidisciplinary residential rehabilitation for severe obesity, thereby defining high-risk patient profiles and guiding tailored retention strategies.

**Methods:**

We retrospectively followed 1,851 adults with obesity discharged from a multidisciplinary residential programme between 2015 and 2018 (median BMI 42 kg m⁻²). Dropout, defined as more than twelve months without contact, was studied with discrete-time survival models; time to rehospitalisation was analysed with Cox regression.

**Results:**

Within twelve months 1,513 patients (87%) discontinued follow-up. Each five-year increase in age lowered drop-out risk (HR 0.97, 95% CI 0.94–0.99, *p* = 0.004); diabetes had a similar protective effect (HR 0.89, 0.79–1.00, *p* = 0.0455). Rehospitalisation occurred in 591 patients (32%). Risk increased with age (5-years increment; HR = 1.05, 95% CI 1.01–1.09, *p* = 0.0191), baseline BMI (HR = 1.04, 95% CI 1.03–1.05, *p* < 0.0001), diabetes (HR = 1.22, 95% CI 1.02–1.30, *p* = 0.0306) and eating disorders (HR = 1.48, 95% CI 1.07–2.05, *p* = 0.0193).

**Discussion:**

Maintaining the benefits of residential rehabilitation is important. In our cohort, 87% of patients dropped out of follow-up within one year and 32% were readmitted. Two distinct profiles emerged: younger and non-diabetic subjects were prone to dropout, while patients with higher BMI, diabetes, or eating disorders were at higher risk of rehospitalization. Early identification of these groups may suggest flexible, technology-assisted follow-up for working-age patients and integrated metabolic-psychiatric care for complex cases, safeguarding outcomes and optimizing resources.

**Supplementary Information:**

The online version contains supplementary material available at 10.1007/s40618-025-02708-z.

## Introduction

Obesity emerges as a multifaceted and complex disorder, distinguished by an excessive accrual of adipose tissue, which precipitates considerable health and wellness risks. It is now recognized as a distinct pathological condition. As delineated by the World Health Organization (WHO), a body mass index (BMI) exceeding 30 Kg/m^2^ qualifies as obesity, escalating to “severe” at a BMI surpassing 40 Kg/m^2^. The global prevalence of obesity has escalated to epidemic dimensions, affecting over 650 million adults as of 2016 [1]. This epidemiological scenario exhibits pronounced regional disparities, with Europe and Italy documenting a consistent uptick in obesity rates over recent decades, mirroring a global trend [2–4].

The American Association of Clinical Endocrinologists (AACE) and the European Society of Endocrinology (ESE) have promulgated comprehensive guidelines, offering evidence-based recommendations for diagnosis, follow-up, and treatment [5, 6]. These guidelines advocate a holistic approach, integrating dietary modifications, physical activity, pharmacotherapy, and surgical interventions, where applicable. In the Piedmont region, in collaboration with the Italian Auxological Institute and our laboratory taking the lead, a Diagnostic Therapeutic Assistance Path (PDTA) for obesity has been established. This protocol encompasses hospitalization within a Metabolic Rehabilitation Centre, followed by an outpatient continuum of care, which may necessitate rehospitalization.

Residential rehabilitation for patients with obesity remains a relatively rare approach, yet it offers distinct advantages. This inpatient program not only enables a comprehensive diagnostic workup, but also supports the early identification of cardiovascular risk and the timely optimization of lipid levels, blood pressure, and the management of diabetes and its complications. Moreover, it enables the early detection and treatment of skeletal, respiratory, and cardiac complications. Employing an interdisciplinary strategy, the program significantly enhances overall health outcomes. Additionally, it initiates an improvement in physical performance, complemented by psychological or psychiatric support for managing eating disorders [7]. The effectiveness of this residential rehabilitation approach is underscored by a marked reduction in BMI and improvements in various anthropometric and biochemical indices. Although the approach is cost-intensive, the long-term benefits are contingent upon patients’ adherence to the program post-discharge to sustain the improvements achieved during hospitalization [8].

A multidisciplinary residential rehabilitation program for severe obesity is not widely available, resulting in scant data within the literature regarding the consequences or predictors of program dropout following admission. In a recent prospective study involving 178 severely obese patients, researchers aimed to identify predictors of dropout from follow-up programs, both with and without a period of residential rehabilitation. They reported a high overall dropout rate of 68.5% at one year. However, an analysis of various predictors (including BMI, waist circumference, body fat percentage, blood glucose, triglycerides, cholesterol, and urate levels) revealed no significant differences in predicting dropout rates at 12 months. Notably, a higher BMI was associated with increased dropout at six months, while elevated uric acid levels were predictive of dropout at two months [9].

This study aims to describe the clinical and sociodemographic characteristics of patients who either complete or discontinue the care pathway, with the goal of identifying predictors of successful outcomes.

## Subjects and methods

### Study design and setting

This study was designed as a retrospective cohort study based on data gathered for the FUOBAUXO project, promoted by the Obesity Centre of Piancavallo Hospital, Italy, IRCCS Istituto Auxologico Italiano. The Obesity Centre of Piancavallo represents a European benchmark for the treatment and rehabilitation of obesity in adults, children and adolescents. Our rehabilitation program begins with an initial assessment phase in General Medicine, followed by a tailored rehabilitation regimen. This initial phase involves a comprehensive clinical metabolic assessment at the Internal Medicine department. It includes estimating the 10-year cardiovascular risk, assessing glucose metabolism, examining hormonal profiles, and exploring other potential contributors to obesity. For patients with diabetes, appropriate screenings for complications are conducted as part of the Diagnostic Therapeutic Assistance Plan, adhering to the guidelines of the Italian National Health System. This plan incorporates bioimpedance analysis, calorimetry, the 6-minute walk test, the hand-grip test, and psychological evaluations using the Psychological General Well-Being Index questionnaire and the Moynihan questionnaire, with all assessments repeated upon patient discharge.

### Participants and eligibility criteria

All patients discharged from the Obesity Centre from May 1, 2015, to December 31, 2018, were eligible to be included in the cohort. Inclusion criteria were age greater than 18 years, BMI > 30 kg/m^2^, and at least one year of follow-up. Patients who opted for bariatric surgery, as well as the few who chose to personally finance their obesity drugs (GLP-1 analogues), were excluded from the study. In Italy, although these drugs are approved, they are not covered by the National Health System; therefore, patients must bear the entire cost themselves.

### Criteria and tools for diagnosis of eating disorders

All participants completed the Italian version of the SCOFF questionnaire at admission. Patients scoring ≥ 2 subsequently underwent a structured clinical interview (Eating Disorder Examination, version 17.0D) conducted by a board-certified psychiatrist. Final eating-disorder diagnoses were established according to DSM-5 criteria (Anorexia Nervosa, Bulimia Nervosa, Binge-Eating Disorder, Other Specified Feeding or Eating Disorder, or Avoidant/Restrictive Food Intake Disorder). Where clinically indicated, additional validated instruments, such as the Eating Disorder Examination-Questionnaire (EDE-Q) or the Binge Eating Scale (BES), were administered to quantify symptom severity and monitor change over time.

### Baseline assessment

Following the initial assessment, patients underwent physiatric and psychological evaluations to customize the motor and physical rehabilitation programs. Depending on individual needs, this could include initiating psychoeducational groups or providing individual psychological support throughout the hospital stay. Dietary and nutritional assessments were also conducted to personalize diet therapy, and in cases of suspected obstructive sleep apnea syndrome (OSAS), nocturnal saturation tests or polysomnography were performed.

### Dietary intervention

In this phase standardized diet therapy was set up by calculating baseline metabolism through the Mifflin equation and considering a caloric deficit of about 15%. All patients were provided with a balanced, hypocaloric Mediterranean diet, comprising three daily meals. The macronutrient distribution included 18–20% protein, 27–30% fat (with less than 8% as saturated fat), 50–55% carbohydrates (with less than 15% as simple sugars), and 30 g of fiber sourced from fresh vegetables.

### Rehabilitation protocol

The subsequent phases of rehabilitation were specifically tailored based on the individual patient’s characteristics and needs. The program included various specialised interventions, if appropriate, such as cardiac, pneumological, physiatric, osteoarticular, and metabolic rehabilitation. If deemed necessary, the patient was enrolled into the specialised support program for bariatric surgery, integrated into our Institute, where he or she received comprehensive information on the potential benefits and risks associated with the procedure. In addition, he or she was meticulously guided through the preoperative evaluation process to ensure optimal preparation and informed decision-making. These services, fully funded by the Italian National Health System, were provided free of charge, ensuring comprehensive and accessible care for all patients. The program specifically targets individuals with grade II-III obesity and associated metabolic complications. These included uncontrolled diabetes, uncontrolled hypertension, hepatosteatosis accompanied by changes in hepatic cytonecrosis markers, and skeletal problems such as osteoarthritis that impaired physical performance in daily activities, as well as respiratory or cardiac complications. These patients were identified as potentially benefiting from an integrated multidisciplinary inpatient pathway.

### Follow-up schedule

The project aimed to study a cohort of obese patients after their hospitalization for weight loss treatment, recording anthropometric and clinical covariates at scheduled control visits. The average period of hospitalization was 28 days, and the first control visit was scheduled three months after discharge while the following were scheduled every four months.

### Outcomes and data recovery

Two different outcomes were considered: dropout from the weight loss program and rehospitalisation for weight loss treatment at the Obesity Centre during the follow-up period. The dropout was defined as a difference greater than 365 days between two consecutive visits (considering also the discharge visit) or between the last visit and the end of the study (December 31, 2019, before the COVID-19 pandemic). The dropout date was identified as the date of the last visit attended by the patient. For both outcomes, even though a patient can experience it more than once, we considered only the first occurrence.

Baseline information regarding age, gender, employment status, education level, BMI, blood pressure values (SBP = Systolic Blood Pressure, DBP = Diastolic Blood Pressure), obesity-related pathologies (diabetes, hypertension) and the psychological status of the patient including depression, anxiety, eating disorders (diagnosed or confirmed by a psychiatric specialist according to the criteria of the Diagnostic and Statistical Manual of Mental Disorders, Fifth Edition) were recorded for each patient. Moreover, weight loss achieved during hospitalisation was recorded. These covariates were selected based on literature and clinical evaluations.

### Statistical analysis

Categorical variables were reported as counts and percentages and the quantitative ones with mean and standard deviation (when normally distributed) or median and interquartile range. The probability of dropout was estimated using a Discrete Time Survival Analysis model (DTSA) following the methodology proposed by Sosu et al. This model is well suited when modeling survival rates with longitudinal categorical data (e.g. dropout). The procedure enables to estimate if an event has occurred and when it has occurred (e.g., a visit in which they dropped out), rather than a simple focus on whether the event has occurred [10]. Hazard risk trajectory and the corresponding survival curves of dropout were obtained by applying an unconditional DTSA without covariates. To estimate the relationship between covariates and the probability of dropout we included all covariates in the DTSA model. Whereas the association between the covariates and the probability of rehospitalisation was evaluated through the implementation of a Cox proportional-hazard (PH) model. The association estimates were reported as hazard ratio (HR) and relative 95% confidence intervals (95% CI).

All statistical tests were two-sided and a p-value less than 0.05 was considered significant. All analyses were performed using SAS 9.4.

## Results

This section delineates the findings of our analyses, focusing initially on patient dropout and subsequently on rehospitalisation.

### Patient dropout

**Fig. 1:**
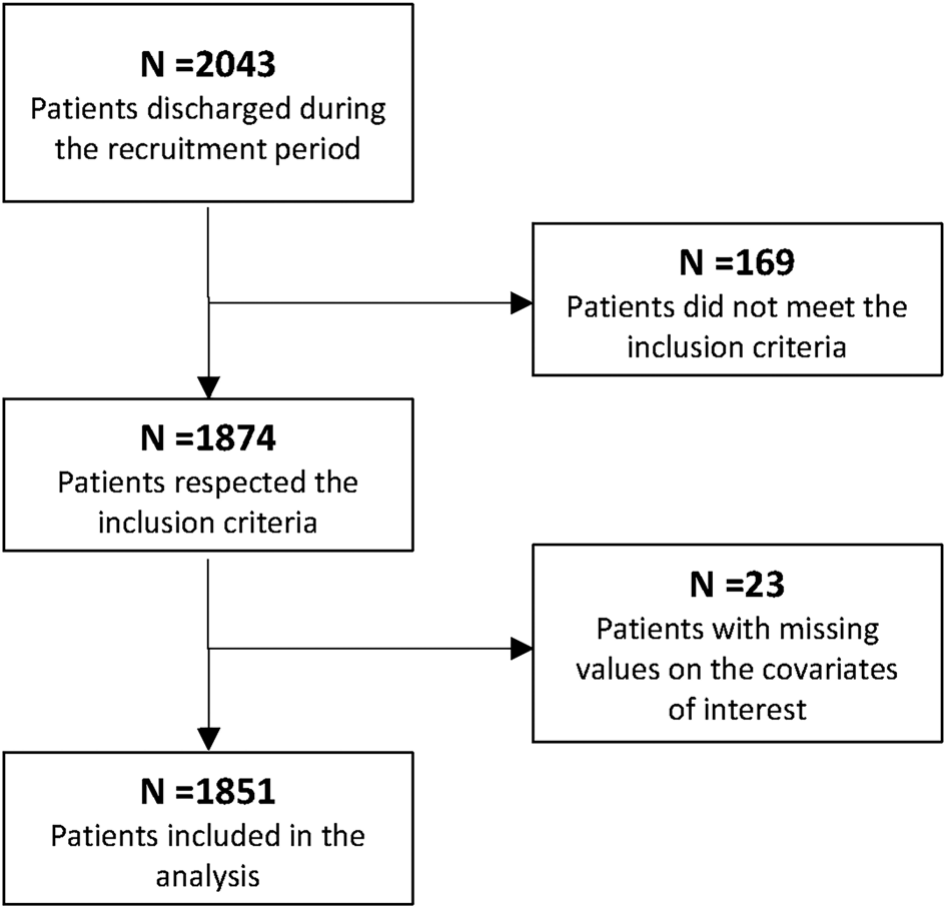
Flow-chart for patients selection

2043 patients were discharged during the recruitment period. 169 of them did not meet the inclusion criteria and 27 presented missing values on covariates of interest (Fig. 1). Therefore, 1851 patients, of whom 1513 (87%) were classified as dropouts, were analyzed. Table 1 reports the sociodemographic and clinical characteristics of the final cohort and separately of dropout and non-dropout patients. The median age of the final cohort was 59 years, with 61% of females and a median BMI equal to 42 kg/m^2^.

**Table 1:** Clinical and sociodemographic characteristics of patients included in the cohort stratified for the outcome variable “dropout”

Variable	All cohort (*N* = 1851)	Dropout (*N* = 1513)	Non dropout (*N* = 338)	*P* value
**Female**,** n (%)**	1120 (61%)	919 (61%)	201 (59%)	0.6652**
**Weight Loss**,** median [range IQ]**	5.1 [3.5–7.1]	5.1 [3.5-7]	5.15 [3.8–7.4]	0.2878*
**SBP**,** median [range IQ]**	140 [130–150]	140 [130–150]	140 [130–150]	0.7139*
**DBP**,** median [range IQ]**	80 [80–90]	80 [80–90]	80 [80–90]	0.9427*
**BMI**,** median [range IQ]**	42 [38.31–47.03]	41.87 [38.16–46.97]	42.15 [38.97–47.32]	0.1678*
**Age**,** median [range IQ]**	59 [50–68]	59 [49–68]	61 [52–69]	0.0244*
**Employed**,** n (%)**	755 (41%)	614 (41%)	141 (42%)	0.7012**
**High education level**,** n (%)**	767 (41%)	641 (42%)	126 (37%)	0.0860**
**Diabetes**,** n (%)**	566 (31%)	442 (29%)	124 (37%)	0.0070**
**Hypertension**,** n (%)**	1191 (64%)	968 (64%)	223 (66%)	0.4882**
**Anxiety**,** n (%)**	380 (21%)	318 (21%)	62 (18%)	0.2710**
**Depression**,** n (%)**	111 (6%)	89 (6%)	22 (7%)	0.6609**
**ED**,** n (%)**	138 (7%)	106 (7%)	32 (9%)	0.1193**

*Wilcoxon test

**Chi-square test

Dropout patients were statistically significantly younger (p-value = 0.0244) and less frequently affected by diabetes (p-value = 0.007). No statistical difference was observed for gender, employment status, level of education, BMI, weight loss during the hospitalization period, blood pressure values, and psychological status.

**Fig. 2:**
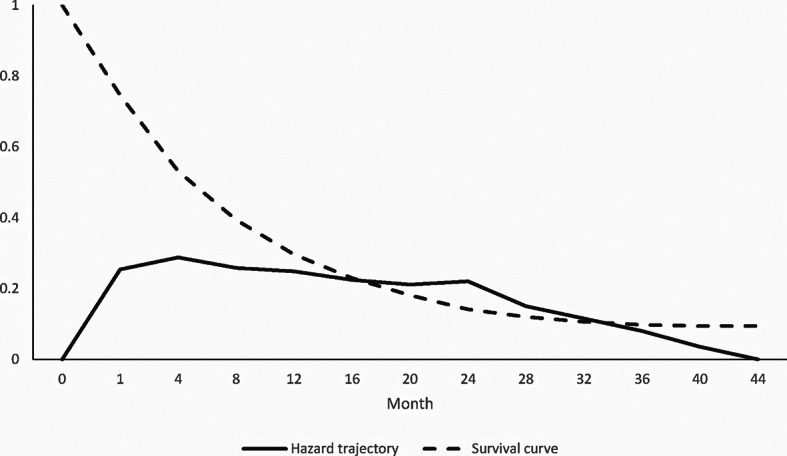
Trajectory of the hazard of dropout and corresponding survival curve. (The x axis reports the months in which patients attend to the visits (month = 0 and month = 1 refer to hospitalization and discharge, respectively); whether, the y axis reports the hazard and survival probability.)

The survival analysis, reported in Fig. 2, reveals that the dropout hazard trajectory after an initial increment until the first follow-up visit decreases slightly. Indeed, a significant proportion of dropouts occurs already at discharge (approximately 25%) suggesting that one patient out of four does not attend to control visits for at least one year. Also, at the first scheduled visit, the survival probability is approximately 53%.

To verify the association between sociodemographic and clinical covariates with the hazard of dropout we fitted a DTSA model. The relative results are reported in Table 2. A 5-year increase in age (HR = 0.97, 95% CI 0.94–0.99, p-value = 0.0040) and the presence of diabetes (HR = 0.89, 95%CI 0.79-1.00, p-value = 0.0455) were statistically and negatively associated with the outcome. Also, depressive status (HR = 1.14, 95% CI 1.00-1.31, p-value = 0.0579) was positively associated with dropout although without statistical significance.

**Table 2:** Results of DTSA model for the outcome “dropout” and of the variable selection performed with the Stepwise method

Variable	HR (95% CI)	*p*-value
**Female**	1.03 (0.91–1.16)	0.6207
**Age**	0.99 (0.98–0.99)	0.0026
**Employed**	0.96 (0.86–1.08)	0.5212
**High Education Level**	1.03 (0.92–1.14)	0.6346
**Weight loss**	0.99 (0.98–1.01)	0.8045
**BMI**	0.99 (0.98-1.00)	0.2261
**SBP**	1.00 (0.99–1.01)	0.2894
**DBP**	1.00 (0.99–1.01)	0.9444
**Diabetes**	0.88 (0.78-1.00)	0.0503
**Hypertension**	1.02 (0.90–1.15)	0.7377
**Anxiety**	0.85 (0.70–1.06)	0.1456
**Depression**	1.14 (0.99–1.30)	0.0589
**ED**	0.86 (0.69–1.07)	0.1698
*Stepwise variable selection*		
**Age**	0.99 (0.99-1.00)	0.009
**Diabetes**	0.89 (0.79–0.99)	0.045

### Rehospitalisation

Table 3 shows the sociodemographic and clinical characteristics of the final cohort when considering rehospitalisation as the primary outcome. Of the 1851 patients included, 591 (32%) were hospitalized a second time at the Obesity Centre. Re-hospitalized patients were statistically significantly older (p-value = 0.0101), with higher values of BMI (p-value < 0.0001), and were also less employed (p-value = 0.0146). In addition, they were affected more by obesity-related diseases like diabetes (p-value = 0.0045) and hypertension (p-value = 0.0039). Lastly, they showed a higher weight loss during the hospitalization period (p-value < 0.0001).

**Table 3:** Clinical and sociodemographic characteristics of patients included in the cohort stratified for the outcome variable “re-hospitalization”

Variable	All cohort (*N* = 1851)	No (*N* = 1260)	Yes (*N* = 591)	*P* value
**Female**,** N (%)**	1120 (61%)	776 (62%)	344 (58%)	0.1654**
**Weight Loss**,** median [range IQ]**	5.1 [3.5–7.1]	4.9 [3.4–6.75]	5.4 [3.9–7.7]	< 0.0001*
**SBP**,** median [range IQ]**	140 [130–150]	140 [130–150]	140 [130–150]	0.2161*
**DBP**,** median [range IQ]**	80 [80–90]	80 [80–90]	80 [80–90]	0.5811*
**BMI**,** median [range IQ]**	42 [38.31–47.03]	41.09 [37.74–46.04]	43.46 [40.04–49.62]	< 0.0001*
**Age**,** median [range IQ]**	59 [50–68]	59 [49–68]	61 [52–69]	0.0101*
**Employed**,** N (%)**	755 (41%)	538 (43%)	217 (37%)	0.0146**
**High Education Level**,** N (%)**	767 (41%)	531 (42%)	236 (40%)	0.3681**
**Diabetes**,** N (%)**	566 (31%)	359 (28%)	207 (35%)	0.0045**
**Hypertension**,** N (%)**	1191 (64%)	783 (62%)	408 (69%)	0.0039**
**Anxiety**,** N (%)**	138 (7%)	91 (7%)	47 (8%)	0.5770**
**Depression**,** N (%)**	380 (21%)	246 (20%)	134 (23%)	0.1178**
**ED**,** N (%)**	111 (6%)	69 (5%)	42 (7%)	0.1684**

*Wilcoxon test

**Chi-square test

The association between sociodemographic and clinical covariates with the hazard of second hospitalization was assessed using a Cox PH model, whose results are reported in Table 4. A 5-year increase in age (HR = 1.05, 95% CI 1.01–1.09, *p* = 0.0191), higher values of BMI (HR = 1.04, 95% CI 1.03–1.05, *p* < 0.0001), diabetes (HR = 1.22, 95% CI 1.02–1.30, *p* = 0.0306), and the presence of eating disorders (HR = 1.48, 95% CI 1.07–2.05, *p* = 0.0193) were statistically and positively associated with second hospitalization.

**Table 4:** Results of Cox PH model for the outcome “re-hospitalization” and of the variable Selction performed with the Stepwise method

Variable	HR (95% CI)	*p*-value
**Female**	0.90 (0.76–1.09)	0.2899
**Age**	1.01 (1.00-1.02)	0.0218
**Employed**	0.90 (0.75–1.07)	0.2308
**High Education Level**	1.04 (0.88–1.24)	0.657
**Weight loss**	1.01 (0.99–1.04)	0.3232
**BMI**	1.04 (1.03–1.05)	< 0.0001
**SBP**	1.00 (0.99–1.01)	0.7378
**DBP**	1.00 (0.98–1.01)	0.4413
**Diabetes**	1.20 (1.01–1.44)	0.0434
**Hypertension**	1.08 (0.90–1.31)	0.4137
**Anxiety**	1.21 (0.88–1.66)	0.2312
**Depression**	1.15 (0.93–1.42)	0.1859
**ED**	1.47 (1.06–2.03)	0.0211
*Stewise variable selection*		
**Age**	1.01 (1.00-1.02)	0.002
**BMI**	1.04 (1.03–1.05)	< 0.0001
**Diabetes**	1.24 (1.04–1.50)	0.015
**ED**	1.48 (1.07–2.03)	0.02

Determining the dropout rate of this program is crucial, particularly in relation to the outcomes linked to proper adherence. The patients included in our cohort exhibited improvements in anthropometric, cardiometabolic, and functional parameters from admission to discharge. This improvement was consistent across both genders, manifesting as reductions in body weight, BMI, waist circumference, and blood pressure, as well as an increase in the distance covered during the 6-minute walk test (as detailed in Table 5 supplementary material).

## Discussion

In our opinion, this study has several strengths. First, it is conducted on a high number (1851 patients) of homogeneous individuals (adults with a median BMI of approximately 40 kg/m2), who were subjected to a standardized care and assistance regime including hospitalization in a specialised facility with a period of residential rehabilitation and a subsequent precise follow-up program. In Italy, this type of intervention is provided and funded by the National Health System, as an alternative to pharmacological treatments or bariatric surgery. Not all patients who meet the updated clinical criteria for clinical obesity proposed by Rubino et al. [11] are willing or eligible for surgery. Furthermore, only a small percentage of patients in Italy can afford the cost of obesity medications. Consequently, our multidisciplinary and integrated residential care program represents a viable treatment alternative, selected by patients from all over the nation. This type of multidisciplinary residential treatment in the inpatient regime is accessible to all Italian patients. Admission is based on a priority scale, which may involve a waiting list. This scale is determined by a team of expert specialists, including internists, endocrinologists, cardiologists, pulmonologists, neurologists and physiatrists. The study offers in-depth revelations on adherence to the proposed program by analyzing clinical, social, and demographic characteristics. Attrition and second hospitalization were assessed as key indicators, defined according to our previous definition.

Our cohort exhibited a slightly higher rate of abandonment after discharge, compared to previously described data in the literature, because: (i) our definition of dropout identified those individuals who had no contact with our institution for an entire year, presumably indicating a definitive interruption of treatment, and (ii) we evaluated the interruption over a long follow up period [9]. This may be attributed to the fact that our Centre serves as a national reference point, attracting patients from across Italy and neighboring countries. It is likely conceivable that accessing the facilities of the Istituto Auxologico Italiano poses challenges for many of our patients, which could influence their ability to continue with follow-up care. The propensity to interrupt treatment is mainly influenced by factors such as age and the presence of diabetes. In particular, increasing age is correlated with a decrease in the probability of abandonment. This observation could be attributed to the relatively simpler logistics that older adults face. This is due to several factors such as retirement from work, the absence of childcare responsibilities, and a generally more flexible schedule, which makes it easier to manage doctor visits in terms of time and organization. Furthermore, older patients likely possess a greater awareness of the fragility of their health, understanding that specialised care can slow the decline in quality of life. The protective role of diabetes on dropout suggests patients’ apprehension regarding the condition, recognizing that weight reduction could improve glucose metabolism, effectively serving as the diabetes management strategy. This dual focus on obesity and diabetes care is perceived as beneficial, underlining the impact of psychological factors, as well as clinical and physical ones, on treatment adherence. Furthermore, at each visit the problems of obesity and diabetes are addressed at the same time, this saves time and increases comfort for the patient. These findings are consistent with literature advocating a multifaceted approach to address obesity treatment adherence challenges [12], suggesting the need of greater personalization.

Identified predictors for a subsequent hospitalization, including age, and underlying health conditions such as diabetes highlight the broad repercussions of obesity on healthcare systems and individuals. These insights are in line with the global escalation of obesity-related complications, which require a proactive and holistic healthcare response [13, 14]. Our study particularly emphasizes the role of BMI and ED as significant predictors of rehospitalization, supporting growing recognition of the intricate relationship between mental health and obesity within clinical practice [14, 15]. Specifically, the likelihood of subsequent metabolic rehabilitation hospitalizations increases by 4% for each unit increase in BMI. This suggests that patients with a higher BMI are more likely to require more than one metabolic rehabilitation hospitalization. Individuals with eating disorders are more likely to be readmitted for residential metabolic rehabilitation treatment, indicating that a single inpatient treatment followed by outpatient follow-up may not be sufficient to effectively address this aspect. Eating disorders such as binge eating disorder often involve (or stem from) deeply rooted psychological issues, such as anxiety, depression, or body image issues, which can take time to address and resolve. Furthermore, some of these individuals may likely need multiple treatment approaches to find the most effective one for their specific needs. Finally, eating disorders are known for their high relapse rate. Daily challenges, stress and other triggers can lead to a relapse, requiring further hospitalizations to restore the patient’s condition. This highlights the complexity of managing these disorders and the need for comprehensive treatment strategies that extend beyond a single hospital stay.

We acknowledge that hazard ratios for age, BMI, diabetes, and eating disorders are relatively close to 1. This reflects their limited independent contribution after adjustment for stronger predictors. However, dropout and, to a lesser extent, readmission are common in our severely obese setting; thus, even small relative differences can translate into a significant operational burden when applied across the population. Moreover, the use of unit-scale increments (per single year of age or per 1 kg/m² in an already obese range) naturally produces estimates close to 1. Grouping by broader categories (e.g., age decades or obesity classes) would increase contrast at the expense of granularity. Overall, our data suggest that these individual-level predictors are unlikely to meaningfully reduce dropout or rehospitalization alone, whereas system-level strategies may be more effective.

Although baseline depressive symptoms did not reach statistical significance as an independent predictor of dropout (HR 1.41, 95% CI 0.94–2.12; *p* = 0.09), the effect size appears clinically relevant. This may reflect symptom alleviation during hospitalization, due to individual psychological support and participation in psychoeducational groups. Prior studies consistently linked depression to early discontinuation in weight management programs [16, 17]. Routine and comprehensive mood screening, along with early psychological intervention, may therefore reduce premature disengagement and improve treatment adherence.

Our investigation, centered on an extensive cohort of individuals with severe obesity (a demographic frequently marginalized in research), illuminates the distinctive challenges and requisites of this group, and integrates a broad spectrum of variables, from socioeconomic factors to psychological states, enhancing the comprehensiveness of our analysis. It offers a comprehensive perspective on the myriad factors that steer patient pathways toward obesity treatment. While numerous studies have ventured into aspects of obesity treatment, little research has investigated the determinants of the interruption of the follow-up program after rehabilitation hospitalization and rehospitalization with the sample size and specifications that our work presents. In particular, several Authors underlined the influence of socio-economic elements on the outcomes of obesity treatment in the past, a concept that our investigation confirms and expands by intertwining clinical and psychological layers [18, 19]. The insights derived from our study have profound implications for the creation of obesity treatment programs. Identifying crucial moments of intervention to avoid treatment abandonment and readmission is fundamental for creating precise support frameworks for people at high risk. Furthermore, the pronounced role of psychological and psychiatric factors, such as eating disorders, requires a comprehensive therapeutic approach that includes the physical and psychological aspects of obesity. We believe that our program should be as inclusive as possible and accessible to everyone. However, we recognize that while multidisciplinary residential rehabilitation is effective, it is also costly. This study was conducted to identify predictors of program dropout in the post-discharge period, which may compromise the benefits gained during hospitalization and negate its advantages. It appears that working-age patients are less likely to maintain follow-up engagements, whereas the presence of diabetic comorbidity appears to enhance adherence to the program. These findings should be considered when selecting patients for residential rehabilitation, especially if prioritization becomes necessary due to waiting lists. Furthermore, a greater personalization of the follow-up program by exploiting digitalized approaches such as telemedicine solutions could help improve adherence in these specific patients. Implementing such criteria could significantly increase the overall effectiveness of the program.

Although, as mentioned, our residential and post-hospital rehabilitation program is accessible to all clinically eligible patients and supported by the Italian National Health System, education and employment emerged as relevant socioeconomic determinants of treatment discontinuation, reflecting broader issues of health inequality. Lower educational attainment was associated with a higher risk of dropout, in line with European data on disparities in obesity care, although the estimate did not reach statistical significance. Financial insecurity, transportation costs, and limited access to paid leave may reduce patients’ ability to complete residential rehabilitation. Policy interventions such as travel vouchers, remote follow-up, and flexible scheduling could improve retention among socially disadvantaged groups.

Despite its contributions, our study is not without limitations. First, its retrospective, single-centre design may introduce selection bias and limit generalizability beyond the Italian healthcare context. Second, the cohort was racially and ethnically homogeneous, preventing assessment of potential disparities in treatment adherence among minority populations. Third, we were unable to retrieve hospitalization events that occurred outside our institutional network and did not systematically detect relevant confounders such as use of psychotropic medications, mobility difficulties, or caregiver burden. These data gaps may have attenuated associations between clinical variables and dropout. Also, the definition of dropout we used was based on clinical experience but was not a universally accepted one. In conclusion, this research contributes significantly to the discourse on obesity treatment, highlighting the complex nature of patient outcomes and the need for tailored interventions. By identifying crucial predictors of treatment interruption and hospital readmission for rehabilitation, we hope to contribute to more effective and integrative strategies for obesity management, to improve patient well-being and healthcare efficiency.

## Supplementary Information

Below is the link to the electronic supplementary material.

Supplementary Material 1

Supplementary Material 2

Supplementary Material 3

## Data Availability

The Raw Data are available for consultation.
