# Supplementary material for: Hazard and determinants of dropout and rehospitalization in patients with obesity after residential rehabilitation
Source: J Endocrinol Invest. 2025 Sep 27;49(1):177–85. doi: 10.1007/s40618-025-02708-z (PMC12847198; doi:10.1007/s40618-025-02708-z)
Supplement: Supplementary file 2 — Supplementary Material 2 [file 40618_2025_2708_MOESM2_ESM.docx]

**Table 1S. Effects of metabolic residential rehabilitation.** **Results of the metabolic rehabilitation program achieved in our cohort of patients, divided into males and females.**

|  | | **Female** | | | **Male** | |
| --- | --- | --- | --- | --- | --- | --- |
|  | | **Mean ± SD** | | | **Mean ± SD** | |
| **Variabile** | **Hospitalization** | | **Discharge** | **Hospitalizaion** | | **Discharge** |
| **Weight (kg)** | 107,10 ± 18,97 | | 102,65 ± 17,88 | 125,08 ± 23,98 | | 118,54 ± 22,46 |
| **BMI (kg/m^2^)** | 43,61 ± 6,94 | | 41,80 ± 6,58 | 42,47 ± 7,01 | | 40,25 ± 6,57 |
| **Waist circ.(cm)** | 120,06 ± 13,13 | | 115,52 ± 12,51 | 130,07 ± 13,90 | | 124,71± 12,77 |
| **LDL cholesterol (mg/dl)** | 124,12 ± 36,77 | | 101,47 ± 33,32 | 114,12 ± 36,68 | | 82,50 ± 32.95 |
| **TRIGLYCERIDES (mg/dl)** | 142,88 ± 62,56 | | 129,33 ± 50,65 | 164,90 ± 77,66 | | 124,57 ± 47,40 |
| **Glycemia (mg/d)** | 113,13 ± 35,21 | | 100,31 ± 18,44 | 122,21 ± 42,11 | | 98,79 ± 19,85 |
| **HBA1C (mmol/mol)** | 45,13 ± 13,71 | | 42,50 ± 9,88 | 42,50 ± 9,88 | | 43,95 ± 11,71 |
| **GAMMA-GT (U/l)** | 28,13 ± 32,13 | | 23,98 ± 30,02 | 46,74 ± 66,27 | | 32,49 ± 34,88 |
| **Systolic pressure (mmHg)** | 138,24 ± 18,74 | | 124,59 ± 12,60 | 139,13 ± 18,75 | | 123,87 ± 11,78 |
| **Diastolic pressure (mmHg)** | 83,09 ± 9,90 | | 76,92 ± 6,78 | 84,33 ± 21,75 | | 76,88 ± 7,19 |
| **6MWT (m)** | 411,93 ± 110,43 | | 441,72 ± 101,32 | 459,29 ± 172,98 | | 493,01 ± 106,17 |
| **TUG (sec)** | 17,44 ± 10,41 | | 15,53 ± 11,75 | 13,53 ± 8,63 | | 11,51 ± 6,70 |
| **SCORE PGWBI** | 60,49 ± 19,50 | | 75,88 ± 17,37 | 71,44 ± 19,21 | | 84,23 ± 16,46 |

TUG: timed up and go (seconds), PGWBI: Psychological General Well-Being Index, 6MWT: 6 minutes walking test (meters walked).
