# Supplementary material for: Hazard and determinants of dropout and rehospitalization in patients with obesity after residential rehabilitation
Source: J Endocrinol Invest. 2025 Sep 27;49(1):177–85. doi: 10.1007/s40618-025-02708-z (PMC12847198; doi:10.1007/s40618-025-02708-z)
Supplement: Supplementary file 1 — Supplementary Material 1 [file 40618_2025_2708_MOESM1_ESM.docx]

Milan, March 18, 2025.

Dear Prof. Pagotto,

Editor-in-Chief,

*Journal of Endocrinological Investigation*

Dear Editor,

We are submitting our manuscript entitled "Determinants of Dropout and Re-hospitalization in Patients with Obesity after Residential Rehabilitation" for consideration by the *Journal of Endocrinological Investigation*. This comprehensive study investigates dropout rates and determinants within a rehabilitation program that integrates in-hospital residential treatment and subsequent follow-up for patients with obesity.

Our research leverages a homogeneous population following a consistent treatment pathway, which enhances the statistical power of our findings and minimizes potential recruitment or data interpretation errors. We explored a range of determinants—clinical, anthropometric, social, and psychological—yielding robust real-world insights into the management of obesity. With a substantial cohort of 1,851 participants, this research is among the most extensive analyses in this area to date.

The novelty of our work lies in its detailed exploration of various factors influencing dropout rates, such as higher BMI, the presence of eating disorders, and the protective impact of diabetes on patient retention. Our findings underscore the complexity of maintaining patient adherence in obesity treatment programs and advocate for a multifaceted clinical approach to effectively address these challenges.

Given the commitment of the *Journal of Endocrinological Investigation*, among other things, to promoting research on the treatment and management of obesity, we believe that our manuscript is perfectly aligned with the interests and objectives of your readers. We are confident that our study will significantly enrich the field by enhancing the understanding of patient attrition, thus supporting the development of more effective, personalized, and integrated care pathways for individuals with obesity.

The manuscript is original, adheres to your submission guidelines, and has not been published or submitted elsewhere. All co-authors have consented to this submission, and we have declared any potential conflicts of interest.

Thank you for considering our work. We eagerly anticipate the opportunity to contribute to your esteemed journal and are available for any further information or clarification you might require.

Sincerely,

Prof Antonella Zambon,

IRCCS Istituto Auxologico Italiano. Biostatistics Unit.

Piazza dell’Ateneo nuovo 1, 20126, Milan, Italy.

[antonella.zambon@unimib.it](mailto:antonella.zambon@unimib.it)

+39 0264485814.
