# Supplementary material for: Hazard and determinants of dropout and rehospitalization in patients with obesity after residential rehabilitation
Source: J Endocrinol Invest. 2025 Sep 27;49(1):177–85. doi: 10.1007/s40618-025-02708-z (PMC12847198; doi:10.1007/s40618-025-02708-z)
Supplement: Supplementary file 3 — Supplementary Material 3 [file 40618_2025_2708_MOESM3_ESM.docx]

Point-by-point responses to reviewers.

Thanks to the Reviewers for the opportunity to improve our Manuscript, let's proceed with the point by point answers.

Reviewer #1: The manuscript addresses a significant clinical issue concerning dropout and re-hospitalization in patients with obesity following residential rehabilitation. However, several improvements are required before it can be considered for publication.
Comments and suggestions are listed below:

-       The abstract would benefit from more emphasis on key findings and their implications while reducing methodological details

***Thanks to the Reviewer for raising this issue: the comment is constructive and allows us to improve and refine the abstract. The abstract now has a more streamlined “methods” section and emphasizes the key messages of the manuscript; more space has been given to the conclusions.***

-       The manuscript contains several grammatical and typographical errors that should be corrected through comprehensive proofreading (e.g., "whit obesity" should be "with obesity" in the abstract and elsewhere).

***We apologize for the errors that escaped our careful analysis, we have revised the text again and the grammatical and typing errors have been corrected.***

-       I suggest dividing the "Subjects and Methods" section into clearly defined subheadings (e.g., Study Protocol, Statistical Analysis, etc.) to enhance readability and methodological clarity.

***We thank the Reviewer for this suggestion. We have divided the Subjects and Methods section into subsections to make the text more readable.***

-       The manuscript mentions eating disorders as predictors of rehospitalization. Please, specify the diagnostic criteria used to identify eating disorders in the patient cohort.

***Thanks to the Reviewer for raising this important point: a new subsection addressing this issue (Criteria and tools for diagnosis of Eating Disorders) has been inserted in the Subjects and Methods section.***

-       The discussion contains some redundancy. Consider restructuring to avoid repetition.

***Thanks to the Reviewer for this idea that gives us the opportunity to improve the Discussion session. Now the Discussion has been deprived of redundancies and repetitions, complex sentences with many words have been simplified for the benefit of ease of reading.***

-       Figure 2 would benefit from improved labeling and a more detailed explanation in the text.

***Following the reviewer suggestion, we have improved the caption of Figure 2 and better specified the axis labels.***

-       Table 1S (supplementary) is mentioned but not fully integrated into the results section.


Reviewer #2: In this manuscript, the Authors addressed critical aspects of adherence and re-hospitalization in obese patients following a multidisciplinary residential rehabilitation program. The large sample size, standardized treatment setting, and comprehensive evaluation of both clinical and psychosocial variables are commendable. The manuscript fits well within the journal's scope and contributes to the literature by identifying predictors of dropout and readmission—key issues in obesity management.

Major Weaknesses & Suggestions
Language & Style
The English is generally comprehensible but would benefit from professional language editing. Some expressions are overly formal or awkward, e.g., "asserting itself as a bona fide pathological entity", "endeavors to dissect", "swiftly provides tools". I suggest tu simplify and clarify sentence structure to improve readability.

***We thank the Reviewer for the valuable feedback. The manuscript has undergone comprehensive language revision to improve readability and eliminate awkward or overly formal expressions. Specifically, we removed or simplified phrases such as “asserting itself as a bona fide pathological entity”, “endeavors to dissect”, and “swiftly provides tools”. We revised the text to use more natural, concise, and accessible scientific language throughout.***


In Table 2 and 4, confidence intervals and HRs for key variables (age, BMI, diabetes, ED) are very close to the null value (HR = 1). The authors should comment more explicitly on the clinical significance of these findings beyond statistical significance.
legend should be corrected.

***We thank the reviewer for this observation that allows us to reason on this important aspect. We have inserted a dedicated paragraph in the Discussion. The new text explains that the hazard ratios for age, BMI, diabetes and eating disorders are around one because their independent contribution becomes modest once the main predictors are corrected. We also believe that the unit scale used (one year of age or 1 kg/m² in an already obese range) naturally produces estimates close to 1. Although these values imply little influence on the individual patient (for example, a HR = 1.05 represents only a relative change of 5%), the high rate of abandonment (and rehospitalization) in this population means that even small relative differences can translate into relevant effects in our specific context (homogeneous population with severe obesity). This interpretation is now included in the discussion.***

Definition of Dropout
The definition used (no visits >365 days apart) is reasonable but should be justified more explicitly and compared with alternative definitions from the literature.

***We thank the reviewer for the comment. In the literature, the commonly considered time points for dropout are 6 or 12 months [Perna et al .2022]. However, the definition typically used refers to the failure to attend the visit scheduled at that specific time point, and does not necessarily reflect an actual discontinuation of treatment. In contrast, our definition identifies as dropouts those individuals who had no contact with our institution for an entire year.***

***Perna S, Salman M, Gasparri C, Cavioni A, Faliva MA, Mansueto F, Naso M, Patelli Z, Peroni G, Tartara A, Riva A, Petrangolini G and Rondanelli M (2022) Two, Six, and Twelve-Month Dropout Rate and Predictor Factors After a Multidisciplinary Residential Program for Obesity Treatment. A Prospective Cohort Study. Front. Nutr. 9:851802. doi: 10.3389/fnut.2022.851802***

Role of Depression
Depression trends toward significance in dropout but is not statistically significant. Given its clinical relevance, the authors should discuss this finding in greater depth, including possible measurement limitations or underdiagnosis.

***We thank the Reviewer for highlighting the role of depression. The Discussion section has been expanded accordingly and 2 relevant bibliographic references have been inserted. Although baseline depressive symptoms did not reach conventional statistical significance (HR 1.41, 95% CI 0.94–2.12; p = 0.09), we believe the effect is clinically relevant. Its magnitude may have been attenuated by the standardized hospital setting and the individual and group psychoeducational counseling sessions provided as part of our multidisciplinary rehabilitation program.***

Impact of Socioeconomic Factors
Although education and employment are included, the discussion could be expanded to integrate findings into broader health inequality contexts, especially considering accessibility to long-term care.

***We appreciate the reviewer's suggestion and have expanded the discussion accordingly. In our sample, participants with lower educational attainment and/or unstable employment showed a higher, but not statistically significant, risk of discontinuation. Barriers to accessibility such as transportation costs, loss of wages, and limited availability of paid leave may partly explain this trend. We now highlight potential health policy actions, such as travel vouchers, tele-follow-up, and flexible hours, that could improve retention among socioeconomically disadvantaged patients.***

Figure Presentation
Figures and tables are clear but would benefit from standardized formatting and titles. For example, clarify axes and legends in Figure 2.

***As suggested by the reviewer, which we want to thank, we have standardized the format and the titles for the tables and clarified the axis of Figure 2.***

Limitations
The authors acknowledge retrospective design and lack of external hospitalization data. However, it would also be important to mention the lack of ethnic/racial diversity data, potential missing confounders (e.g., medication use, mobility issues), and generalizability outside Italy.

***We thank the reviewer for this valuable observation and have rewritten the “Limitations” section more comprehensively. We now emphasize that the retrospective and monocentric nature of the study may introduce selection bias and limit the transferability of our findings outside the Italian healthcare context. We also acknowledge that our cohort is racially and ethnically homogeneous, which precludes any analysis of potential disparities in treatment adherence among minority populations. Furthermore, we emphasize that we were unable to capture hospital admissions outside our institutional network and did not systematically record potential confounders such as use of psychotropic or weight-loss medications, mobility limitations, or caregiver burden, factors that could have attenuated the observed associations. By explicitly addressing these issues, we believe that the section now offers a more transparent interpretation, thanks to these changes.***

Minor Comments
Abstract: Typo in line "patients whit obesity" → should be "with".
Abstract: The semi-colon in "drop-out;." should be removed.

Consider rewording "patients undergoing a comprehensive treatment program including..." → too wordy; "patients enrolled in a comprehensive residential and outpatient rehabilitation program" would suffice.

References: Reference 11 to Rubino et al., 2025 seems to lack page numbers

Use consistent tense throughout (e.g., sometimes present, sometimes past).

***We apologize for these errors, which have been corrected in the new version of the Manuscript.***
